# Holistic nursing care practice and associated factors among nurses in public hospitals of Wolaita zone, South Ethiopia

**DOI:** 10.1186/s12912-023-01517-0

**Published:** 2023-10-18

**Authors:** Selamawit Ataro Ambushe, Nefsu Awoke, Birhanu Wondimeneh Demissie, Tiwabwork Tekalign

**Affiliations:** 1 School of Nursing, College of Health Science and Medicine, Wolaita Sodo University, Wolaita Sodo, Ethiopia; 2 School of Nursing and Midwifery, College of Health Science, Addis Ababa University, Addis Ababa, Ethiopia; 3 School of Nursing & Midwifery, Faculty of Health, University of Technology Sydney, Sydney, Australia; 4 School of Nursing, College of Medicine and Health Science, Arbaminch University, Arbaminch, Ethiopia

**Keywords:** Holistic nursing practice, Holistic care, Comprehensive care, Ethiopia

## Abstract

**Background:**

Holistic nursing care is an approach to patient care that takes into account the physical, social, spiritual, and psychological needs of the patient. Providing holistic care has been found to be an effective way to prevent diseases and death, as well as improve the quality of healthcare provided to patients. However, despite its perceived benefits, many nurses lack experience with holistic care and only focus on patients’ physical needs, treating them as biological machines while ignoring their spiritual, mental, and social needs. Therefore, this study aimed to assess the practice of holistic nursing care and its associated factors among nurses.

**Methods:**

A hospital-based cross-sectional study was conducted among 422 sampled Nurses working in public hospitals in Wolaita Zone. Systematic random sampling was applied to select the study participants. A self-administered, pretested questionnaire was used to collect the data. The collected data were entered into Epi Data version 4.6 and analyzed using SPSS version 25. Binary and multivariable logistic regression analyses were used to identify factors associated with holistic nursing care practice. Statistical significance was declared at a p-value less than 0.05. The strength of the association was indicated by the AOR and 95% CI.

**Result:**

The study found that the overall practice of holistic nursing care was 21%. Nurses with a diploma in nursing (AOR: 0.28; 95% CI: 0.11, 0.71), nurses working in a hospital with no continuous in-service learning (AOR: 0.39; 95% CI: 0.20, 0.76), nurses with a poor relationship with patients (AOR: 0.31; 95% CI: 0.16, 0.58), and nurses with poor knowledge of holistic nursing care practice (AOR: 0.41; 95% CI: 0.21, 0.7) were factors associated with a lower likelihood of practicing holistic nursing care.

**Conclusion:**

This study found that the practice of holistic nursing care among nurses working in public hospitals in the Wolaita Zone was low. Level of education, the unit of work, continuous in-service learning, the nurse-patient relationship, and the knowledge of nurses were factors associated with holistic nursing care. The provision of in-service training and the creation of trusting, positive relationships were suggested to improve the practice of holistic nursing care.

## Background

Holistic nursing care (HNC) is an important aspect of nursing practice in that it emphasizes the care of the patient as a whole [1]. The American Nursing Association (ANA) defines holistic care as an integration of body, mind, emotion, spirit, sexual, cultural, social, energetic, and environmental principles and modalities to promote health, increase well-being, and actualize human potential [2]. Holistic health care is complete care that contemplates the physical, social, spiritual, emotional, and economic needs of the patient, his or her response to illness, and the effect of the illness on the ability to meet self-care needs [3].

The goals of holistic nursing are centered around improving health, reducing suffering, and preventing illness. Holistic nurses focus on protecting, promoting, and optimizing health and wellness. They also strive to provide support to individuals in finding peace, comfort, and balance during times of illness [4]. Globally, there is a continuously increasing need for holistic nursing care. Because there is a direct relationship between quality of life and holistic care, any conceptualization of quality of life that is made should be holistic to the extent that, at any given time, different aspects of a person’s life will influence other aspects [3, 5].

The practice of holistic care is an effective way to prevent diseases and death as well as improve the quality of health care provided to patients [6]. So that more people around the world are turning away from conventional medicine in favor of holistic health care. In the United States, one out of every three people seeks holistic care [7]. While HNC enhances patient satisfaction by improving the quality of care [8], its absence increases treatment costs, lengthens hospital stays, and increases the risk of developing new complications [1].

On the other hand, nurses in many hospitals do not practice holistic treatment. According to previous surveys, 67% of patients in the United States do not receive holistic care, but only 5% of patients in Germany do. Research shows that the elimination of holistic care poses a crisis in the nursing profession because it aids in the promotion and restoration of health [9, 10].

Furthermore, data reveals that nurses are inexperienced with holistic care, neglect the holistic model of care, do not employ the holistic technique, and only examine patients’ corporeal requirements, treating them as biological machines while ignoring their spiritual, mental, and social needs [11, 12].

Nurses’ practice of holistic care can be assumed to have a major impact on patients’ outcomes and patient safety. Hence, there is potential to improve the quality of care and patient safety by enhancing nurses’ practice of holistic care. Interventions aiming to enhance nurses’ practice of holistic nursing care need to target the factors that are important for holistic nursing care [13].

Factors such as inadequate time, experience, motivational and organizational issues, and a lack of resources can all prevent holistic care from being practiced [1]. In addition, evidence suggests that most nurses do not practice holistic care because they were educated in the biomedical allopathic system, which focuses on disease rather than people, and because they lack a thorough understanding of holistic nursing care [11, 12].

Because of a profound limitation in routine treatment, Africans, including Ethiopians, are looking for ways to better enhance their health needs by using other alternatives [14]. All components of the World Health Organization’s public health model will be addressed by effectively integrating holistic care for patients and their families [5]. Despite its perceived benefit, there has been no study conducted in Ethiopia on holistic nursing care to determine the extent of the practice and its associated factors. Therefore, this study was aimed at assessing the practice of holistic nursing care and its associated factors in Wolaita Zone public hospitals.

## Methods

### Study setting

The study was conducted at public hospitals in the Wolaita Zone. There are 8 governmental hospitals (one comprehensive specialized hospital and 7 primary hospitals) and 68 health centers in the Wolaita zone. There are 3,790 health care providers in Wolaita Zone, including 1,656 nurses, 197 general practitioners, 357 laboratory technicians, 602 midwives, 460 health officers, 62 anesthesiologists, 24 radiology professionals, 360 pharmacy professionals, 28 emergency surgeries, and 44 different types of specialists [15].

### Study design and period

A Hospital-based cross-sectional study design was applied. The study was conducted from May 1 to June 30, 2022.

### Population

The study included all nurses who are permanent recruits working in public hospitals in the Wolaita zone and those who were involved in the direct care of admitted patients working in the medical, surgical, gynecology, pediatric [16], ophthalmology, dialysis, oncology, psychiatry, neonatal intensive care unit (NICU), orthopedic, and post-anesthesia care units, and the intensive care unit. The study excluded nurses who were critically ill, those who worked in the OPD, and nurse managers who had no direct patient contact.

### Sample size determination

The sample size was calculated using a single population proportion formula with an estimated practice of 50% (because there was not the same study conducted with the same objective in our country). With a 5% marginal error (d) and a confidence interval of 95% (Z α/2 = 1.96). Based on these assumptions, the sample size was calculated using the following formula:

1 $$n = \frac{{{{({z_{\alpha /2}})}^2} \cdot pq}}{{{d^2}}}$$

The final calculated sample size after adding 10% non-respondents was **422**.

### Sampling techniques

There are eight government hospitals in the Wolaita zone. The total number of nurses who were involved in direct patient care in each hospital was, 480 in WSUCSH, 45 in Humbo Primary Hospital, 37 in Bodit Primary Hospital, 30 in Kindo Didaye Primary Hospital, 39 in Bitena Primary Hospital, 50 in Gesuba Primary Hospital, 42 in Bele Primary Hospial, and 55 in Bombe Primary Hospitals. A proportional size allocation was applied to each hospital, and we get a sample of 268 from WSUCSH, 25 from Humbo Primary Hospital, 21 from Bodit Primary Hospital, 16 from Kindo Didaye Primary Hospital, 22 from Bitena Primary Hospital, 28 from Gesuba Primary Hospital, 23 from Bele Primary Hospial, and 29 from Bombe Primary Hospitals. The nurses involved in direct patient care at each hospital were listed in a series of orders to create a sampling frame. Then a systematic random sampling technique with a sampling interval of 2 for each hospital was used to get the study participants, and random start 1 was determined by lottery.

### Data collection tools, procedures and data collectors

A self-administered, structured questionnaire was used to collect the data. The questionnaire was prepared by reviewing different literature [1, 17]. Twenty-two questions, divided into four domains (physiological, psychological, sociocultural, and spiritual), were used to evaluate holistic nursing care practice. The participants’ knowledge level was measured by eight measuring items. Knowledge level was scored by the proportion of correctly answered items. The mean was calculated, and those who answered above the mean were regarded as having good knowledge, and respondents who answered knowledge-related questions below the mean value were regarded as having poor knowledge [17]. The condition of the patient was described based on American Hospital Association guidelines [18]. A total of eight BSc nurses’ and four supervisors with MSc qualifications were involved in the data collection process. The data collectors distribute the questionnaire after explaining the purpose and technique of filling it out, and then collect the questionnaire back.

### Operational definitions

#### Good practice in each domain

The respondent who answered a practice-related question in the given domain above the mean [17].

#### Poor practice in each domain

The respondent who answered a practice-related question in the given domain below the mean [17].

#### Good holistic nursing care practice

The respondent who fulfilled all domains of holistic nursing care practice [19, 20].

#### Poor holistic care practice

The respondent who missed at least one domain of holistic care practice [19, 20].

**Good knowledge**: The respondent who scored above the mean [17].

#### Poor knowledge level

The respondent who scored below the mean [17].

#### Good relationship

Respondents who answered the relationship-related question above the mean value [1].

#### Poor relationship

Respondents who answered the relationship-related question below the mean value [1].

#### Job satisfaction

When the total score for the job satisfaction subscale was greater than the computed mean, they were satisfied with the overall aspect of their work [21].

**The condition of the patient** Was described based on the American Hospital Association guidelines as.

- **Good**: Vital signs are stable and within normal limits. The patient is conscious and comfortable. Indicators are excellent.
- **Fair**: Vital signs are stable and within normal limits. The patient is conscious but may be uncomfortable. Indicators are favorable.
- **Serious**: Vital signs may be unstable and not within normal limits. The patient is acutely ill. Indicators are questionable.
- **Critical**: Vital signs are unstable and not within normal limits. The patient may be unconscious. Indicators are unfavorable [18].

### Data quality control

To maintain the quality of the data, two days of training were given to data collectors and supervisors by the principal investigator on the data collection tools and procedures. A pre-test was conducted on 5% of the sample size before actual data collection in Tercha General Hospital, Dawuro Zone. The reliability of the tool was checked by calculating a Cronbach’s Alpha of 89.7%. Close follow-up and supervision were conducted during the data collection period. The collected data were checked by supervisors every day.

### Data analysis

The collected data was checked for its completeness, then coded, and the data was entered using Epi Data version 4.6 and analyzed using SPSS version 25. Descriptive statistics were used to describe each individual variable using the frequency distribution, percentage, mean, and standard deviation. A binary logistic regression analysis was computed for each predictor variable in holistic nursing care. Then variables with a p value less than 0.25 were entered into the multivariable logistic regression analysis. Both crude odd ratios (COR) and adjusted odd ratios (AOR) with 95% CI were used to identify predictor variables. Variables that had a p-value less than 0.05 were considered significant.

### Ethical consideration

The research was approved by the Ethical Review Committee of Wolaita Sodo University College of Health Science and Medicine. An official letter was written to each hospital director. All methods were carried out in accordance with the Declaration of Helsinki and its later amendments or comparable ethical standards. All study participants were encouraged to participate in the study, and at the same time, the data collectors told the participants that they had the right not to participate. Finally, data was collected after assuring the confidentiality of responses and obtaining written informed consent from the study participants.

## Results

### Socio-demographic characteristics

A total of 391 participants were included in the study, with a response rate of 92.65%. The majority of respondents, 220 (56.3%), were male, and one hundred eighty-one (46.3%) of the nurses were in the age category between 25 and 30 years, with a mean ± SD age of 29 ± 5.9 years. Two hundred fifteen (55%) of respondents were married, and three hundred seven (78.5%) of the nurses had a BSc degree or above, and 237 (60.6%) had less than 5 years of experience, with a mean ± SD of work experience of 5.3 ± 4.3 years. Ninety-nine (25.3%) of the respondents were from the medical ward (Table 1).

**Table 1:** Socio-demographic characteristics of nurses in public hospitals of Wolaita Zone, South Ethiopia, 2022

Variables	Categories	Frequency	Percentage [22]
Sex	Male	220	56.3
	Female	171	43.7
Age (Years)	< 25	110	28.1
	25–30	181	46.3
	31–35	60	15.3
	> 36	40	10.2
Marital status	Single	173	44.2
	Married	215	55.0
	Widowed	3	0.8
Level of Education	Diploma	76	19.4
	BSc Degree or above	315	80.6
Experience (Years)	< 5	237	60.6
	> 6	154	39.4
Ward/unit	Medical	99	25.3
	Surgical	57	14.6
	PACU	14	3.6
	ICU	42	10.7
	Gynecological ward	47	12.0
	Pediatric ward	48	12.3
	Others ^*^	84	21.5

Others ^*^: wards such as ophthalmological, dialysis, oncologic, psychiatric, neonatal intensive care unit and orthopedic wards

### Practice of holistic nursing care

**Fig. 1:**
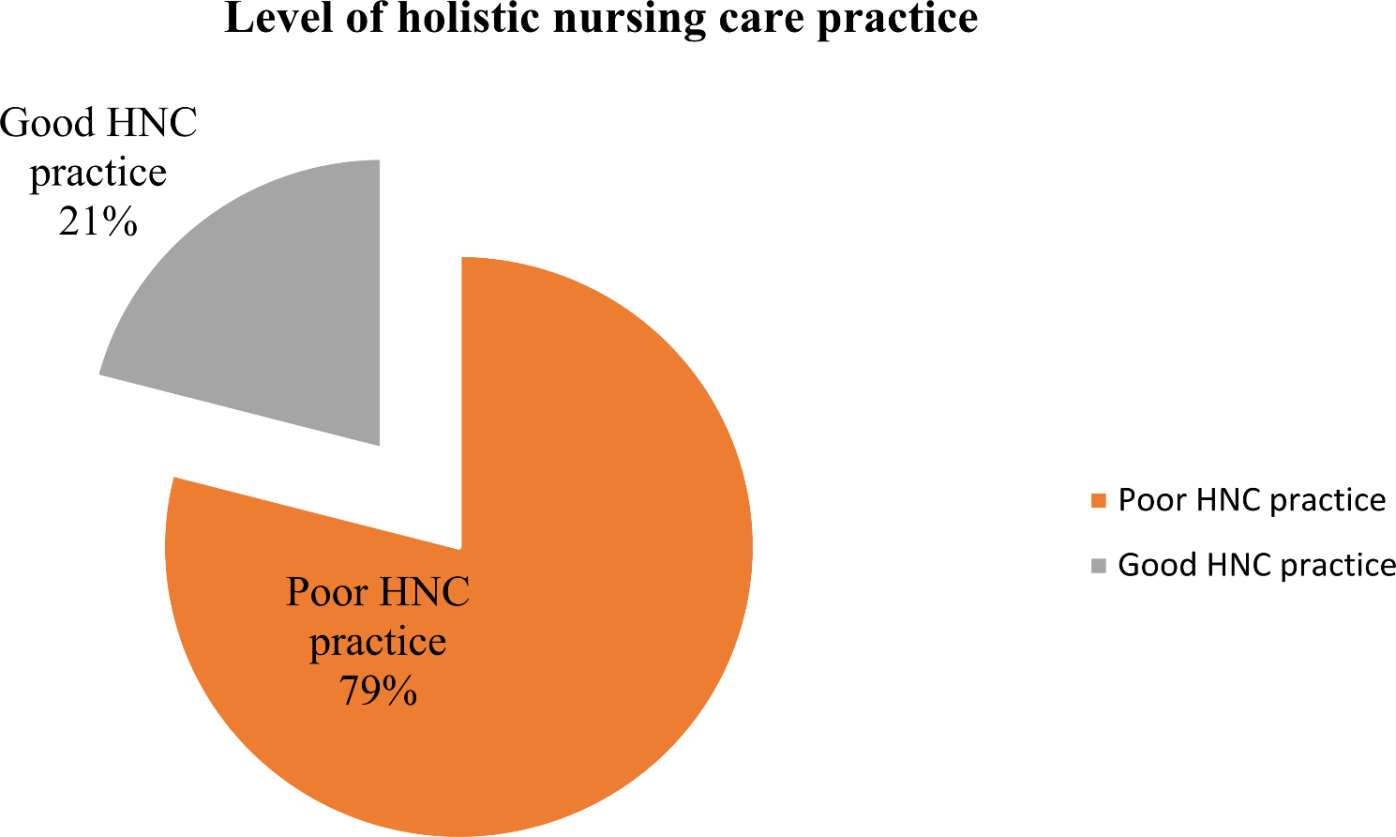
Level of holistic nursing care practice of nurses in public hospitals of Wolaita Zone, South Ethiopia, 2022

The majority, 206 (52.7%) of respondents, had good practice in the physiological dimension; 208 (53.2%) had good practice in the socio-cultural dimension; 211 (54.0%) had good practice in the psychological dimension; and 211 (54.0%) had good practice in the spiritual dimension (Table 2). The overall holistic nursing care practice in all dimensions in this study was 82 (21.0%) (Fig. 1).

**Table 2:** Holistic nursing care practice of Nurses in public hospitals of Wolaita Zone, South Ethiopia, 2022

Variables	Categories	Frequency	Percentage [22]
Physiological aspect	Yes	206	52.7
	No	185	47.3
Psychological aspect	Yes	211	54.0
	No	180	46.0
Socio-cultural aspect	Yes	208	53.2
	No	183	46.8
Spiritual aspect	Yes	211	54.0
	No	180	46.0

### Nurse related factors

Two hundred thirty-nine (61.1%) of the nurses had good knowledge of holistic nursing care. One hundred eighty-seven (47.8%) of the nurses had a good relationship with patients, and 209 (53.5%) were satisfied with their jobs (Table 3).

**Table 3:** Nurse related factors of respondents in public hospitals of Wolaita Zone, South Ethiopia, 2022

Variable	Categories	Frequency	Percentage [22]
Knowledge	Good	239	61.1
	Poor	152	38.9
Nurse Patient relationship	Good	187	47.8
	Poor	204	52.2
Job Satisfaction	Satisfied	209	53.5
	Dissatisfied	182	46.6

### Factors associated to holistic nursing care of the respondents

Ten variables were selected as candidate variables out of 19 independent variables for multiple logistic regression analysis. In multivariable logistic regression, five variables (nurse’s level of education, ward or unit of work, continuous in-service learning, nurse-patient relationship, and knowledge level of nurses) were found to be significantly associated with holistic nursing care practice.

Nurses who had a diploma in nursing were 72% less likely to practice holistic nursing care than nurses who had a BSc. Degree or higher in nursing (AOR = 0.28; 95% CI: 0.11, 0.71). Nurses working in ICU were 78% (AOR = 0.22; 95% CI: 0.07, 0.69); Gynecological wards were 71% (AOR = 0.29; 95% CI: 0.10, 0.82); Pediatric wards were 86% (AOR = 0.14; 95% CI: 0.04, 0.44); and Nurses who were working in other wards (such as ophthalmological, dialysis, oncologic, psychiatric, and orthopedic wards) were 71% (AOR = 0.29; 95% CI: 0.13, 0.65) less likely to practice holistic nursing care compared to nurses who were working in the medical ward.

Nurses who were working in the hospital where there was no continuous in-service learning were 61% (AOR = 0.39; 95% CI: 0.20, 0.76) less likely to practice holistic nursing care compared to those working in the hospital where there was continuous in-service learning.

Nurses who had a poor relationship with patients were 69% (AOR = 0.31; 95% CI: 0.16, 0.58) less likely to practice holistic nursing care compared to those nurses who had a good relationship with patients.

**Nurses who had poor knowledge of holistic nursing care practice were 59% (AOR = 0.41; 95% CI: 0.21, 0.79) less likely to practice holistic nursing care compared to those nurses who had good knowledge** (Table 4).

**Table 4:** Factors associated with Holistic Nursing care practice among nurses in public hospitals of Wolaita Zone, South Ethiopia, 2022

Variables	Category	HNC Practice		COR ( 95%CI)	AOR (95%CI)
		Yes	No		
Age	< 25	18 (22.0%)	92 (20.0%)	1	1
	25–30	38 (46.3%)	143(46.6%)	1.36 (0.73, 2.52)	1.54(0.69, 3.44)
	31–35	15 (18.3%)	45 (14.7%)	1.70 (0.79, 3.69)	2.01(0.76, 5.35)
	> 36	11 (13.4%)	29 (9.5%)	1.94 (0.82, 4.57)	2.98(1.03, 8.56)
Sex	Male	54 (65.9%)	166 (53.7%)	1	1
	Female	28 (34.1%)	143 (46.3%)	0.60(0.36, 1.00)	0.66(0.35, 1.24)
Level of education	BSc Degree and above	73 (89.0%)	242 (78.3%)	1	1
	Diploma	9 (11.0%)	67(21.7%)	0.45 (0.21, 0.94)	0.28 (0.11, 0.71)*
Ward/unit of work	Medical	31 (37.8%)	68 (22.0%)	1	1
	Surgical	13 (15.9%)	44 (14.2%)	0.65(0.31, 1.37)	0.61 (0.25, 1.50)
	PACU	2 (2.4%)	12 (3.9%)	0.37(0.08, 1.73)	0.16 (0.02, 1.05)
	ICU	6 (7.3%)	36 (11.7%)	0.37 (0.14, 0.96)	0.22 (0.07, 0.69)*
	Gynecological	7 (8.5%)	40 (12.9%)	0.38 (0.16, 0.95)	0.29 (0.10, 0.82)*
	Pediatric	5 (6.1%)	43 (13.9%)	0.26 (0.92, 0.71)	0.14(0.04, 0.44)*
	Other	18 (22.0%)	66 (21.4%)	0.60 (0.31, 1.17)	0.29 (0.13, 0.65)*
Condition of patient	Undetermined	6 (7.3%)	20 (6.5%)	1	1
	Good	34 (41.5%)	165 (53.4%)	0.69 (0.26, 1.84)	0.79(0.24, 2.68)
	Fair	15 (18.3%)	35 (11.3%)	1.43 (0.48, 4.27)	3.04(0.72, 12.78)
	Serious	3 (3.7%)	28 (9.1%)	0.36 (0.08, 1.60)	0.69(0.13, 3.80)
	Critical	24 (29.3%)	61 (19.7%)	1.31 (0.47, 3.66)	1.23(0.34, 4.52)
Continuous in-service learning	Yes	56 (68.3%)	165 (53.4%)	1	1
	No	26 (31.7%)	144 (46.6%)	0.53 (0.32, 0.89)	0.39 (0.20, 0.76)*
Availability of nursing process	Yes	63 (76.8%)	214 (69.3%)	1	1
	No	19 (23.2%)	95 (30.7%)	0.68 (0.39, 1.20)	1.47(0.84, 2.59)
Job satisfaction	Satisfied	38 (46.3%)	171 (55.3%)	1	1
	Dissatisfied	44 (53.7%)	138 (44.7%)	1.44 (0.88, 2.34)	0.77(0.42, 1.43)
Nurse-patient relationship	Good relationship	58 (70.7%)	129 (41.7%)	1	1
	Poor relationship	24 (29.3%)	180 (58.3%)	0.30 (0.18, 0.50)	0.31 (0.16, 0.58)*
Knowledge level of nurses	Good knowledge	62 (75.6%)	177 (57.3%)	1	1
	Poor knowledge	20 (24.4%)	132 (42.7%)	0.43 (0.25, 0.75)	0.41 (0.21, 0.79)*

## Discussions

This study revealed that 21.0% (95% CI: 17.6, 25.1) of nurses practiced all the components of holistic nursing care. Nurses level of education, ward or unit of work, continuous in-service learning, nurse-patient relationship, and knowledge level on holistic nursing care were found to be the factors associated with holistic nursing care practice.

Nurses practiced individual components of holistic nursing care; however, the overall holistic nursing care practice of the individual nurse was 21.0% (95% CI: 17.6, 25.1). This might be due to nurses focus on individual components of patients rather than addressing patient needs holistically [11] or the lack of a comprehensive guide to practicing holistic nursing care in hospitals [23]. [23].

The individual components as well as the overall practice of holistic nursing care in our study were lower than in the study conducted in Cameroon, which found that 28.6% of the nurses based their practice on the biological aspect, 18.6% on the psychological aspect, 27.1% on the social aspect, 14.3% on the spiritual aspect, and 11.14% truly practiced all of the aspects [24]. This difference might be due to the socio-demographic differences among nurses or the differences in resources allocated for the health care systems in the two countries.

The study results showed that 61% of nurses had knowledge of holistic nursing care. This finding supported the study conducted in Kenya, which showed that nurses keep their knowledge of holistic care up-to-date but are unable to transfer their theoretical knowledge into the practice of holistic nursing care. This might be due to a lack of in-service training in the provision of holistic nursing care. There must be adequate time, knowledge, motivation, and training given to nurses at all levels of health institutions in order to provide holistic care for patients [1].

It was observed that nurses who had a diploma in nursing were 72% less likely to practice holistic nursing care than nurses who had a BSc. Degree or above in nursing (AOR = 0.282; 95% CI: 0.113, 0.709). This finding is consistent with cross-sectional studies conducted in Malaysia and Turkey [25, 26], [25–27] which conclude that better education and training for nurses are required for satisfying clients and sustaining the outcomes of patient care. This finding is also in line with the former study, which concluded that a lack of education affects clinical practice [28]. This might be due to the fact that nurses with a lower educational level might be less exposed to the concept of holistic nursing care and practice. The information gap might have helped them understand the purpose and importance of HNC, which may have influenced their practice. A lack of opportunity for education can hinder the provision of holistic care [22].

Accordingly, nurses who had worked in a hospital in which there was no continuous in-service learning were 61% less likely to practice holistic nursing care than those who had worked in a hospital in which there was continuous in-service learning (AOR = 0.39; 95% CI: 0.20, 0.76). This finding was consistent with a former study done in Iran, which implied that nurses would provide holistic care if educators provided holistic care in a practical model [11]. The study conducted in India was also consistent with this finding, which resulted in a mismatch between academic learning and clinical performance that can hinder the provision of holistic nursing care [29]. Additionally, the former qualitative study conducted in South Africa indicated that the need for continuous in-service education on different topics had an impact on holistic care [30]. This might be due to the fact that [30] nurses with lower awareness regarding holistic nursing care may not be able to understand the purpose and importance of HNC, which might affect their HNC practice.

Nurses who had a poor relationship with patients were 69% less likely to practice holistic nursing care than those who had a good relationship with patients (AOR = 0.31; 95% CI: 0.16–0.58). This result was in line with the study conducted in Kenyatta’s national hospital, which indicated that the interpersonal relations and rapport between the nurses and the patients can directly affect the holistic care provision [1]. This finding supports a qualitative study conducted in South Africa, which recommended that demonstrating companionship and respect for the patient were important during holistic care [30]. This is also in line with Watson’s theory of human caring, which places emphasis on cultivating and maintaining caring and helpful relationships [31]. The qualitative study also supported this finding by stating that there should be humanity in caring for patients, a desire to maintain helpful relationships, and love and kindness for patients in order to provide holistic nursing care. This might be due to the fact that nurses who are social and emotionally stable are more capable of identifying the needs of patients [29] and providing care accordingly.

Nurses who had poor knowledge of holistic nursing care practice were 59% less likely to practice holistic nursing care than those with good knowledge (AOR = 0.41; 95% CI: 0.21, 0.79). The study conducted in South Africa [30], and Kenya [1], and Saudi Arabia [12] supported this finding. This might be due to an in-depth knowledge of holistic care that increases the nurse’s provision of care, or it might be due to knowledge and evidence used in practice that raises awareness of professional and personal accountability and the dilemmas of practice, which improves care [30, 32]. The mixed-type study done in Kenya was consistent with this finding, which indicated that the knowledge base of the nurses concerning the care they provide can affect the provision of holistic care [1]. Additionally, this finding also supported the study conducted in Saudi Arabia, which concluded that to provide patients with holistic care, nurses must be knowledgeable in the holistic approach [12] .

## Conclusions

Holistic nursing care practice was low among nurses, according to this study. Lower levels of education, the unit of work, a lack of continuous in-service learning, a poor nurse-patient relationship, and the poor knowledge level of nurses on holistic nursing care were factors associated with the practice of holistic nursing care. Therefore, the provision of in-service training is suggested to improve the knowledge and practice of holistic nursing care. Moreover, encouraging and motivating nurses to properly provide HNC for patients should be emphasized in public hospitals.

## Data Availability

The datasets used and/or analyzed during the current study are available from the corresponding author on reasonable request.

## Abbreviations

AOR: Adjusted Odds Ratio
COR: Crud Odds Ratio
HNC: Holistic Nursing Care
ICU: Intensive Care Unit
NICU: Neonatal Intensive Care Unit
PACU: Post anesthesia Care Unit
